# Ingestion of Milk Containing Very Low Concentration of Antimicrobials: Longitudinal Effect on Fecal Microbiota Composition in Preweaned Calves

**DOI:** 10.1371/journal.pone.0147525

**Published:** 2016-01-25

**Authors:** Richard Van Vleck Pereira, Svetlana Lima, Julie D. Siler, Carla Foditsch, Lorin D. Warnick, Rodrigo Carvalho Bicalho

**Affiliations:** 1 Department of Population Medicine and Diagnostic Sciences. College of Veterinary Medicine, Cornell University, Ithaca, NY, United States of America; 2 Department of Population Health and Reproduction. College of Veterinary Medicine, University of California Davis, Davis, CA, United States of America; Colorado State University, UNITED STATES

## Abstract

Although antimicrobial drugs are central to combat disease in modern medicine, the use of these drugs can have undesired consequences for human and animal health. One consequence is the post-therapy excretion of pharmacological agents, such as the elimination of drug residues at very low concentrations in the milk of lactating mammals. Limited information is currently available on the impact from the exposure of the gut microbiota to drug residues using *in vivo* natural models. The objective of our study was to address this knowledge gap and evaluate the effect on the fecal microbiota composition from feeding preweaned dairy calves raw milk with residual concentrations of ampicillin, ceftiofur, penicillin, and oxytetracycline from birth to weaning. At birth, thirty calves were randomly assigned to a controlled feeding trial where: 15 calves were fed raw milk with no drug residues (NR), and 15 calves were fed raw milk with drug residues (DR) by adding ceftiofur, penicillin, ampicillin, and oxytetracycline at final concentrations in the milk of 0.1, 0.005, 0.01, and 0.3 μg/ml, respectively. Fecal samples were rectally collected from each calf once a week starting at birth, prior to the first feeding in the trial (pre-treatment), until 6 weeks of age. Sequencing of the microbial 16S rRNA genes was conducted using the Illumina MiSeq, which provides a high resolution of the microbiota down to the genus level. Discriminant analysis showed that, except for pre-treatment samples, calves fed milk with drug residues and calves fed milk without drug residues easily discriminated at the genus level on their weekly microbial profile. However, analysis comparing the abundance of taxon between NR and DR showed significant differences only at the genus levels, and not at the phylum, class, order or family levels. These results suggest that although drug residues can result in clear discriminate gut microbial communities, they do not result in disruption of taxonomic levels above the genus.

## Introduction

Although antimicrobials are of great importance to both human and animal health, exposure of the microbiota to drugs has been associated with imbalance of the intestinal microbiota followed by harmful effects to the host, such an invasion by pathogenic bacteria and impacts on nutrient absorption by the host [1,2]. In dairy cow, gastrointestinal dysbiosis has been shown to be associated with clostridial disease by *Clostridium botulinum* [3]. This shows the importance of the gut microbiota to the host, protecting against enteropathogens [4,5], extracting nutrients and energy from the diet [6,7], and contributing to normal immune function [8]. Most current studies available using high throughput sequencing approaches to study the exposure of the microbiota to drugs have focused on the impacts of therapeutic concentrations of antimicrobial drugs. However, a growing concern is the impact of involuntary daily exposure of the microbiota to antimicrobial drugs at very low concentrations, such as from drinking water or ingesting foods containing drug residues.

An undesired consequence from the use of antimicrobial drugs in human and veterinary medicine is the elimination of pharmaceutical residues and/or metabolites in waste (feces and urine), or in milk of lactating mammals. These drug residues are environmental pollutants that can increase drug resistance and could potentially affect the balance of the microbiomes exposed to these drugs [9,10]. Due to the large volume of drugs used in food animals, residues eliminated after exposure to antimicrobials is a major public health concern, [11]. In addition to livestock sources, antimicrobial agents have been detected in water (e.g. rivers, drinking water) [12,13], aquacultures (e.g. shrimp farms) [14], irrigation of plants [15], and in soil and sediments [16].

In humans, administration of drugs to pre or post-partum mothers can result in ingestion by infants of milk containing drug residues [17]. In the United States 32.7% of deliveries are cesarean sections, and because cesarean section is the single most important risk factor for postpartum maternal infection in humans, it is usually preceded and/or followed by treatment with antimicrobial drugs [18,19]. Limited information is currently available on the impacts from the ingestion of milk containing drug residues on the microbiota of infants, however administration of antimicrobials at therapeutic doses to infants has been identified as a risk factor for asthma, allergy and obesity later in life, and has been suggested to be linked to the disruption of the microbiota [20–22].

In the dairy industry, most antimicrobial drugs used to treat cows result in the milk from these animals being withheld from sale because of the presence of drug residues above the tolerance concentration established by the U.S. Food and Drug Administration (FDA). A tolerance level is a concentration determined by the FDA at which residues of a substance present in a food will have no harmful effects on the human consumers of the food product. To make use of the nutrient value of waste milk, 33% of dairy farms in the United States feed preweaned calves milk containing drug residues [23].

Preweaned calves are regarded as monogastric animals because they have a physically and functionally different GI system from that of the mature ruminant and, similar to human infants, their diet is mainly composed of milk until weaning [24]. In a recent study conducted by our research group, milk withheld from sale and fed to calves was collected from several dairy farms in central New York and screened for drug residues [25]. The three most common drug residues identified were the following β-lactams: penicillin G, and ampicillin and the cephalosporin drug ceftiofur. Commercially available β-lactams drugs are commonly used in lactating dairy cattle to treat conditions such as mastitis, respiratory disease and metritis. Cephalosporin drugs are of particular interest because they are commonly used in human medicine, one example being ceftriaxone, a broad spectrum antimicrobial commonly used to treat neonates for gonococcal ophthalmia, invasive salmonellosis in children, and as a second line drug for sepsis and meningitis.

As in human research, limited information is available on the impact of feeding preweaned dairy calves drugs at concentrations typically found in milk withheld from sale on the composition of the microbiota, and no study has used a controlled feeding trial to fully evaluate this issue. The objective of our study was to address this knowledge gap and evaluate the effect on the fecal microbiota composition of feeding preweaned dairy calves raw milk with residual concentrations of ampicillin, ceftiofur, penicillin, and oxytetracycline from birth to weaning.

## Materials and Methods

### Ethics statement

Fecal samples were collected from calves (*Bos taurus*) that were housed on Cornell University facilities. The research protocol was reviewed and approved by the Institutional Animal Care and Use Committee of Cornell University (Protocol number: 2012–0090).

### Study design and sample collection

Randomized non-blinded controlled feeding trials were conducted at the College of Veterinary Medicine, Cornell University (Ithaca, NY, USA) from June 2013 to March 2014. Three feeding trials were completed with a total of 10 male calves in each trial, with 5 calves belonging to each treatment group. All thirty calves enrolled in the trials were purchased from a local dairy farm and enrolled in the study on their date of birth. Control calves (n = 15) were enrolled in the trials concurrently with test calves (n = 15). At least one author in the study was involved in all calf collection at the farm. Upon collection, a physical examination was performed and calves were weighed. Additionally, the assignment of calves to study groups was done at the farm by pairing calves born on the same day by weight and using a coin toss to randomly allocate a calf as either a test or control. Once a calf was assigned to a treatment group it received an identification tag, which was placed in the right ear. All calves were fed 2–4 liters of maternal colostrum from individual cows within the first 4 h of life. Colostrum fed to calves in both milk feeding treatments originated from the dairy farm where calves were collected. At this farm cows were under the same management. After feeding of colostrum, calves were transported from the source farm to Cornell University.

Calves were individually housed in concrete box stalls to prevent contact between calves. Blood samples were collected from each calf in the first 24–48 hours of life, and the serum total protein was measured to assess adequacy of passive transfer. Control calves were fed raw milk without the addition of antimicrobial drugs (NR), and test calves were fed raw milk with the addition of low concentrations of ceftiofur, penicillin, ampicillin and oxytetracycline (DR). All calves were bucket fed one gallon of raw whole milk twice a day from birth to 6 weeks of age. Feedings occurred once in the morning and once in the afternoon with approximately 12 hour intervals between feedings. A non-medicated pelleted calf starter (18% crude protein, 3% crude fat, 8% crude fiber; DuMOR Calf Starter, Tractor Supply Co., Brentwood, TN) was offered from day 7 until day 42 of life up to a maximum of 1 kg/day. To prevent cross-contamination between calves, each calf stall had dedicated equipment and supplies, and all study personnel used personal protective equipment when entering each calf stall, which was changed after exiting the calf stall. No calves required treatment with therapeutic antimicrobial drugs during the trial.

Single-use gloves were used to collect rectal fecal samples from each calf once a week starting at birth, prior to the first feeding in the trial (pre-treatment), until 6 weeks of age. Fecal samples were stored for 9 to 17 months at -20°C until DNA extraction [26].

### Spiking milk with drug residues

Raw milk used to feed calves was collected daily from the Cornell University College of Veterinary Medicine Dairy Farm. Antimicrobial stock solutions used to spike milk were prepared one week prior to each calf trial. Stocks were prepared by diluting powdered drugs in distilled water to a concentration of 100 μg/mL for ampicillin sodium salt, 1,000 μg/mL for ceftiofur sodium, 50 μg/mL for penicillin G sodium, and 3,000 μg/mL for oxytetracycline hydrochloride. Individual sterile cryovials with 2.28 ml of each stock solution for each antimicrobial drug were stored at -80°C until used [27–29]. Individual antimicrobial stocks (instead of a cocktail of drugs) for each antimicrobial drug were prepared to avoid potential interactions between different antimicrobials before adding it to the milk.

Milk containing drug residues fed to DR calves was prepared twice a day 10 to 20 minutes prior to feeding. For each drug, a tube containing 2.28 ml of antimicrobial stock solution was thawed at room temperature and added to a batch of 22.8 liters of raw milk which was stirred for 1 minute at approximately 400 RPM prior to feeding to calves. The final concentration of each antimicrobial drug in the milk fed to DR calves was calculated to be: 0.01 μg/ml of ampicillin sodium, 0.1 μg/ml of ceftiofur sodium, 0.005 μg/ml of penicillin G sodium, and 0.3 μg/ml of oxytetracycline hydrochloride. Because limited information is currently available on the impacts of waste milk on selection of resistance, drugs were pooled in the milk (instead of tested individually) to simulate what is expected to be observed on dairy farms. Milk fed to NR calves was also stirred for 1 minute at approximately 400 RPM prior to feeding, but without the addition of any antimicrobial and by using clean dedicated equipment to avoid drug contamination from milk fed to DR calves. After each feeding, equipment was thoroughly cleaned separately by milk feeding treatment group using a dedicated brush and hot water, liquid soap, and sodium hypochlorite (The Clorox Co., Oakland, CA). The selection of drugs and the concentration added to the milk was based on an article published by our research group where we screened waste milk withheld for sale at dairy farms in central New York [25]. In that study the most prevalent drugs detected by LC-MS/MS were ceftiofur (mean ± SE concentration = 0.151 ± 0.042 μg/mL), penicillin G (mean ± SE concentration = 0.008 ± 0.001 μg/mL), and ampicillin (mean ± SE concentration = 0.472 ± 0.43 μg/mL). In addition, one sample had detectable concentrations of oxytetracycline (0.01 μg/mL). Because of the high frequency use of this drug in dairy cattle, it was also added to the milk fed to DR calves [30]. Moreover, the tolerance and safe levels for drug residues in raw milk used for human consumption determined by the Federal Department of Agriculture (FDA) were also used to determine at what concentration the selected antimicrobial drugs should be added to the milk.

Raw milk used in the trial was screened daily for drug residues prior to its use in the study. Two commercial tests were used: New SNAP Beta-lactam Test Kit (IDEXX Laboratories Inc., Westbrook, ME) which detects penicillin (LOD = 0.005 ppm), ampicillin (LOD = 0.01), amoxicillin (LOD = 0.01 ppm), cephapirin (LOD = 0.02 ppm), and ceftiofur (LOD = 0.1 ppm) residues in raw milk; and SNAP Tetracycline Test Kit (IDEXX Laboratories Inc., Westbrook, ME) which detects tetracycline (LOD = 0.05 ppm), oxytetracycline (LOD = 0.05 ppm), and chlortetracycline (LOD = 0.1 ppm) residues in raw milk. During the entire trial all milk samples from the dairy farm tested negative for drug residues.

Milk spiked with antimicrobial drugs fed to DR calves was screened weekly for drug residues using the New SNAP Beta-lactam Test Kit and the SNAP Tetracycline Test Kit. As expected, all spiked milk samples tested positive. There are no current commercial test kits for detecting drug residues in cow colostrum. Additionally the commercial kits available for milk are not appropriate for colostrum because the high fat percentage and viscosity of colostrum compared to milk can interfere in the results of the test. Therefore colostrum fed to calves was not screened for drug residues. However, colostrum fed to calves in both milk feeding treatments originated from one dairy farm where cows were under the same management.

### DNA extraction

DNA was extracted from fecal samples using the Mobio PowerSoil DNA isolation kit (MO BIO Laboratories) with slight modifications based on previously published protocols [31]. Approximately 50 ng of feces was thawed at room temperature and transferred to the bead-beating tube, which was heat treated at 65°C for 10 minutes and then 95°C for 10 minutes. Bead-beating of the samples was performed for 5 minutes in a Mini-Beadbeater-8 (Biospec Products, Battersville, OK, USA). The remaining DNA extraction procedure followed the standard protocol supplied by the company. DNA was extracted from all fecal samples within 1 month time period, and within 2 weeks DNA samples were PCR amplified for 16S rRNA genes.

### PCR amplification of the V4 hypervariable region of bacterial 16S rRNA genes

The 16S rRNA gene was amplified by PCR from individual metagenomic DNA samples of feces using barcoded primers. For amplification of the V4 hypervariable region of the bacterial/archaeal 16S rRNA gene, primers 515F and 806R were used according to a previously described method optimized for the Illumina MiSeq platform [32]. The earth microbiome project (http://www.earthmicrobiome.org/) was used to select 140 different 12-bp error-correcting Golay barcodes for the 16S rRNA PCR, as previously described [32,33]. The 5'-barcoded amplicons were generated in triplicate using 3μl DNA template, 1× GoTaq Green Master Mix (Promega, Madison, WI), 1 mM MgCl2, and 10 μM of each primer. The PCR conditions for the 16S rRNA gene consisted of an initial denaturing step of 94°C for 3 min, followed by 35 cycles of 94°C for 45 s, 50°C for 1 min, and 72°C for 90 s, and a final elongation step of 72°C for 10 min. Replicate amplicons were pooled and purified with a QIAquick PCR Purification Kit (Qiagen, Valencia, CA, USA), and visualized by electrophoresis through 1.2% (wt/vol) agarose gels stained with 0.5 μg/ml ethidium bromide before sequencing. Blank controls in which no DNA was added to the reaction were also conducted. Purified amplicon DNA was quantified using the Quant-iT™ PicoGreen® dsDNA Assay Kit (Life Technologies Corporation, Carlsbad, CA, USA).

### Sequence library analysis and analysis of data

Amplicon aliquots of fecal samples were standardized to the same concentration and then pooled into 2 different runs (one run with 139 samples and a second run with 71 samples) according to individual barcode primers for the 16S rRNA gene. Final equimolar libraries were sequenced using the MiSeq reagent kit v2 (300 cycles) on the MiSeq platform (Illumina, Inc., San Diego, CA, USA). The obtained 16S rRNA gene sequences were processed through the open source software pipeline Quantitative Insights Into Microbial Ecology (QIIME) version 1.7.0-dev [34]. Sequences were filtered for quality using established guidelines [35]. Sequences were binned into Operational Taxonomic Units (OTUs) based on 97% identity using UCLUST [36] against the Greengenes reference database [37], May 2013 release. Low-abundance clusters were filtered and chimeric sequences were removed using USEARCH [36]. The representative sequences for each OTU were compared against the Greengenes database for taxonomy assignment, and only full-length, high-quality reads (-r = 0) were used for analysis. Additionally, we generated a species-level OTU table using the MiSeq Reporter Metagenomics Workflow. The MiSeq Reporter classification is based on the Greengenes database (http://greengenes.lbl.gov/) and the output of this workflow is a classification of reads at multiple taxonomic levels: kingdom, phylum, class, order, family, and genus.

Using the obtained OTU information, we evaluated each sample’s richness and diversity using the Chao1 index and the Shannon index, respectively. To evaluate the effect of calf milk feeding treatment over time in weeks on the richness and diversity indexes, multivariate mixed logistic regression models were fitted to the data using the GLIMMIX procedure of SAS. The independent variables treatment group, time in weeks of sampling, and interactions were included in all models. The effect of animal identification nested within trial number was controlled in the models as a random effect. Least square means and standard error of the means for these indexes were obtained using the LSMEANS statement.

The relative abundance of different bacterial taxa in each sample was used as covariates in stepwise discriminant analysis models built in JMP Pro 11. In the discriminant analysis used in our study, taxa were removed in a stepwise manner until only variables with a P value <0.05 were retained in the final model. Multiple discriminant analyses were conducted using the following variables as covariates: time in weeks of sampling by treatment group, treatment group by each week of sampling, and treatment group for all samples collected from calves after receiving the first feeding treatment (weeks 1 to 7). Canonical scores for these analyses were used to create graphical display of the results for taxon in the analyses.

Taxa that were retained after the stepwise manner discriminant analysis for treatment group by discriminating or not by each week of sampling were evaluated in JMP through a screening analysis using the false discovery rate (FDR) to correct for multiple comparisons. The FDR is an approach for multiple testing that aims at controlling the proportion of significant results that in fact are false rejections of the null hypothesis, minimizing type-1 statistical errors. Taxa with a significantly different relative frequency between treatment groups were selected for further analysis using a multivariate mixed logistic regression model in PROC GLIMMIX, as described above.

Descriptive analysis for the comparisons of the average weight gain during the 6 weeks of the calf trial between NR calves and DR calves was conducted in SAS using PROC GLIMMIX.

## Results

### Descriptive data

The mean birth weight for the NR calves was 45.3 kg (range: 31–53 kg) and for the DR calves the mean was 44 kg (range: 34–53 kg). Average weight gain during the 6 weeks of the calf trials was 1.1lbs (95% C.I. 0.9–1.4) for NR calves and 1.35lbs (95% C.I. 1.1–1.6) for DR calves. There was no significant difference in the average weight gain during the 6 weeks of the calf trials between NR calves and DR calves (*P* value = 50.3).

### Sequencing results, core microbiome description, and taxa prevalence

**Fig 1:**
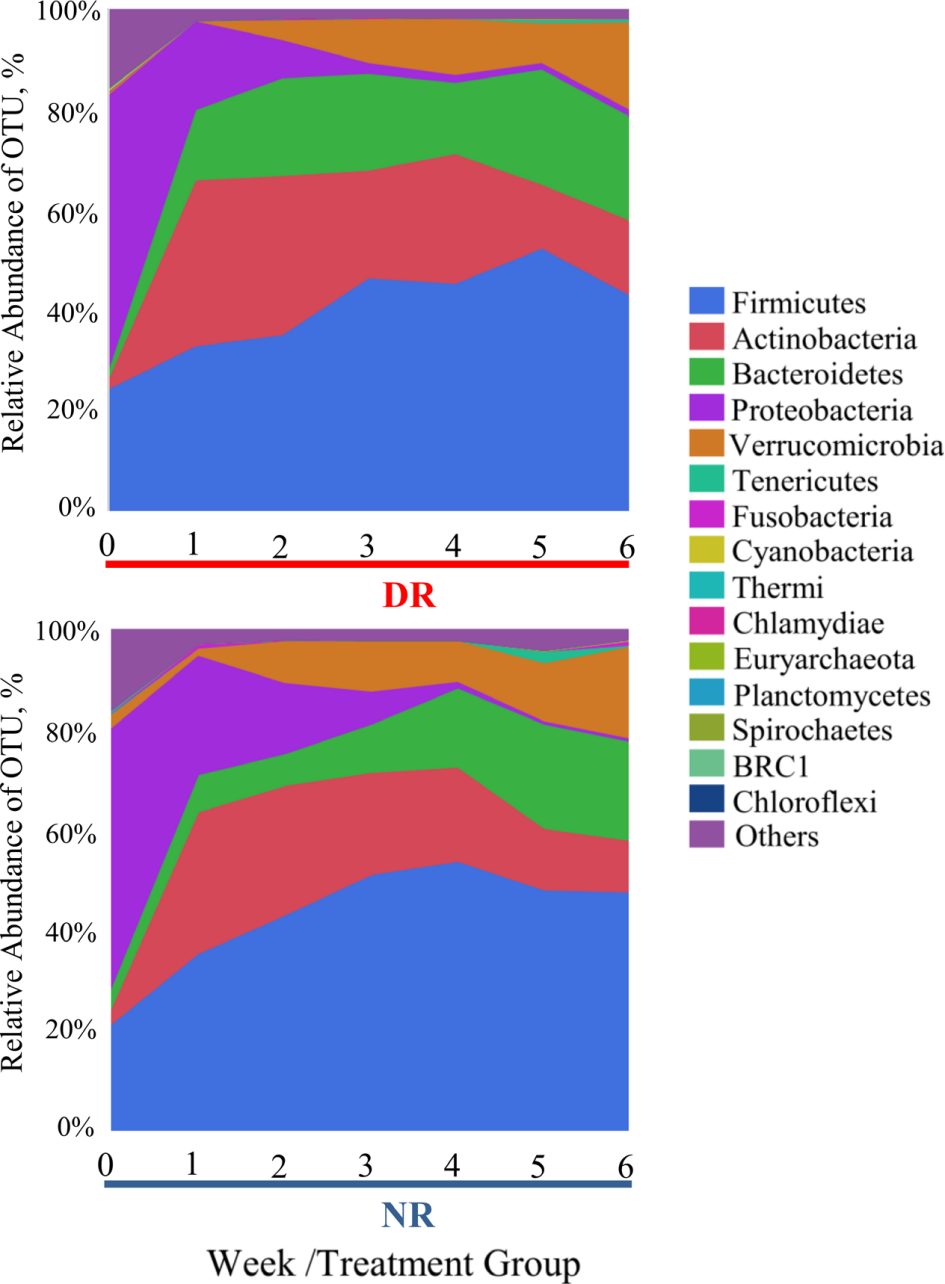
Average percentage of the 16 most prevalent phyla for each treatment group by week of sampling. Week 0 is the sample collected from calves at birth, prior to receiving any treatment.

**Fig 2:**
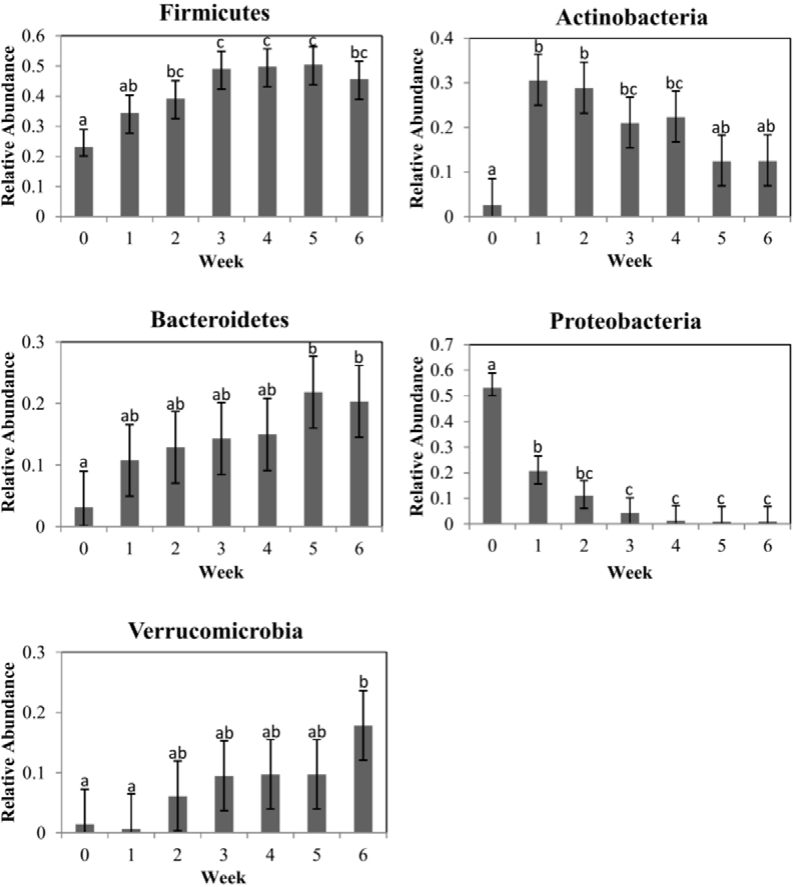
Mean relative abundance for the 5 most common phyla for each sampling week. Data of both treatment groups was combined. **Error bars correspond to a 95% confidence interval.** Different letters (a–c) between weeks within each graph indicate means that are statistically different (P < 0.05). Week 0 is the sample collected from calves at birth, prior to receiving any treatment.

Quality-filtered reads for 16S sequences yielded 18,613,793 sequences in total with a median length of 301 bases per read, and an average coverage of 88,637 sequences per sample. The six most common phyla for both treatment groups were: Firmicutes, Actinobacteria, Bacteroidetes, Proteobacteria, Verrucomicrobia, and Tenericutes (Fig 1). Although no significant difference in phyla relative abundance between treatment groups was observed (S1 Table), when the data of both treatment groups was combined, differences between weekly samples were observed (Fig 2). Of the top 4 phylum, Firmicutes, Bacteroidetes and Verrucomicrobia relative abundance tended to increase in abundance over time, while Proteobacteria tended to decrease in abundance over time. The phylum Actinobacteria had a significant increase in relative abundance from week 1 to week 2, and after week 3 it gradually decreased in abundance.

### Relative abundance of the most prevalent genera in fecal samples

**Fig 3:**
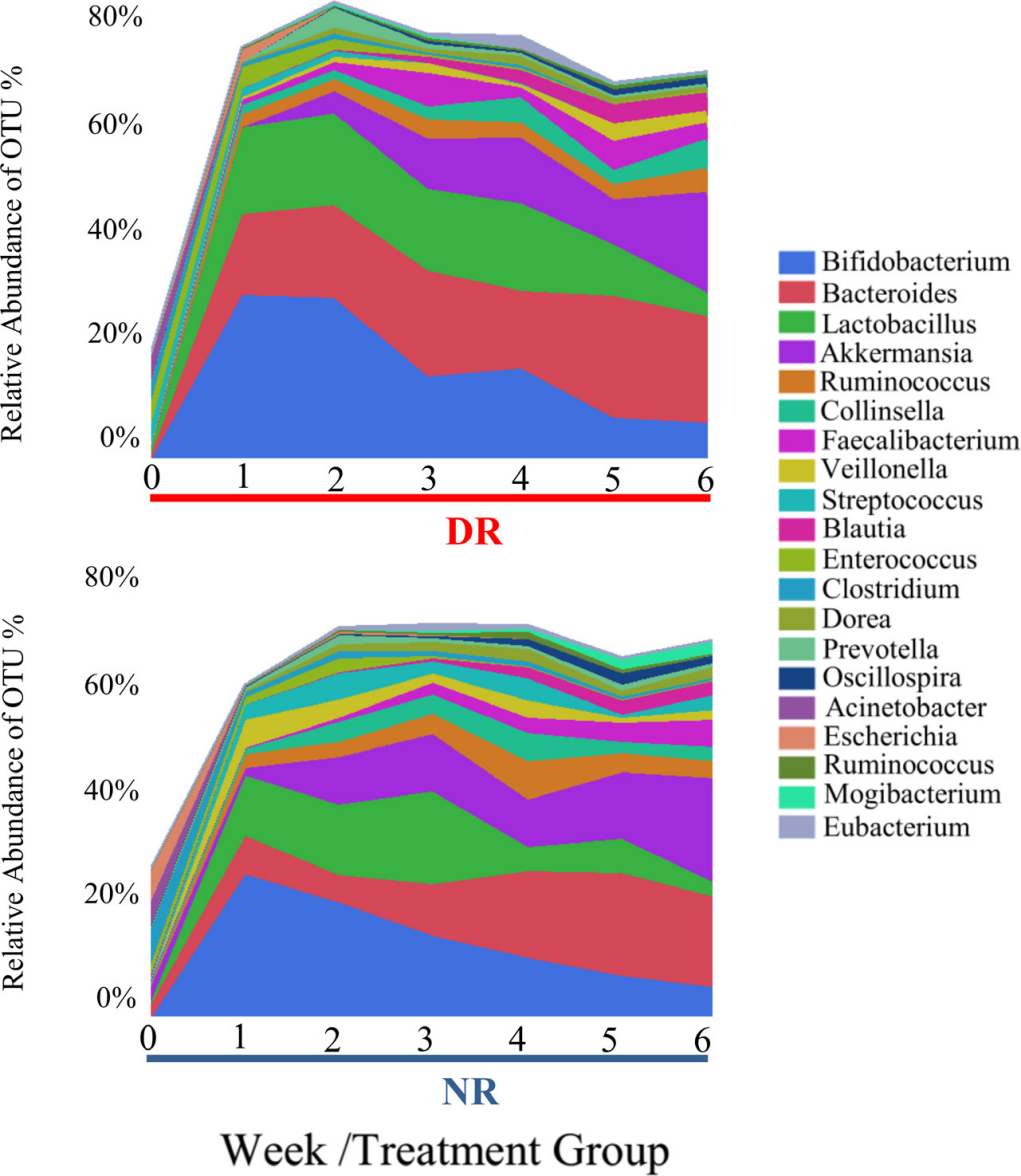
Average percentage of the 20 most prevalent genera for each treatment group by week of sampling. Week 0 is the sample collected from calves at birth, prior to receiving any treatment.

The relative abundance of the 20 most prevalent microbes at the genus level is depicted in Fig 3. Of these microbes, the only one that was significantly different between treatment groups for at least one week in the linear regression analysis was *Veillonella spp* (*P value* = 0.04), for which the prevalence and results from linear regression analysis are displayed on S1 Fig. The results of the discriminant and linear regression analysis conducted on samples collected from calves after receiving the first feeding treatment (data combined from weeks 1 to 7) showed that the relative abundance of *Clostridium spp*. (NR = 0.008 and DR = 0.004; *P* value = 0.03) and *Streptococcus spp*. (NR = 0.02 and DR = 0.004; *P* value = 0.004) was significantly reduced in the DR samples when compared to NR samples.

### Discriminant analysis

**Fig 4:**
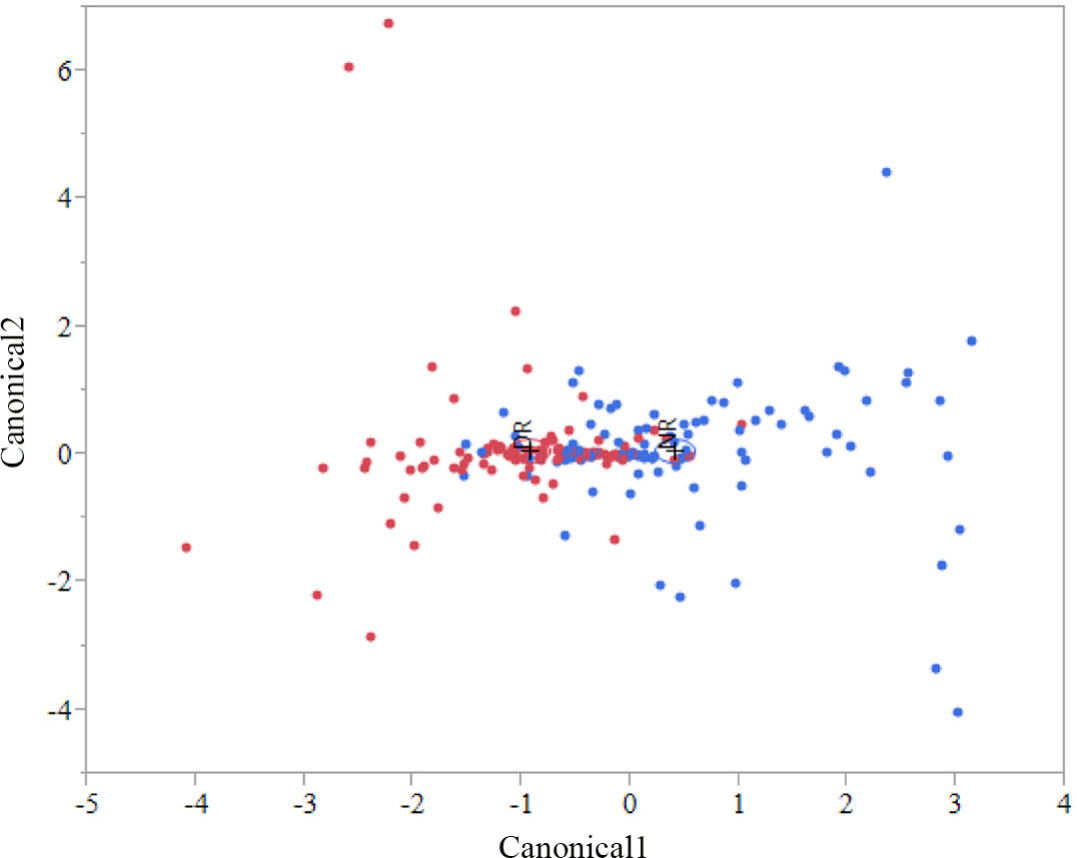
Discriminant analyses of fecal sample microbiome by treatment group for all sampling weeks. Differences in the fecal microbial profiles for each treatment group (NR = blue dots, DR = red dots) are illustrated by Canonicals 1 and 2. Week 0 was not included in this analysis. An ellipse indicates the 95% confidence region to contain the true mean of the group, and a plus symbol indicates the center (centroid) of each group.

**Fig 5:**
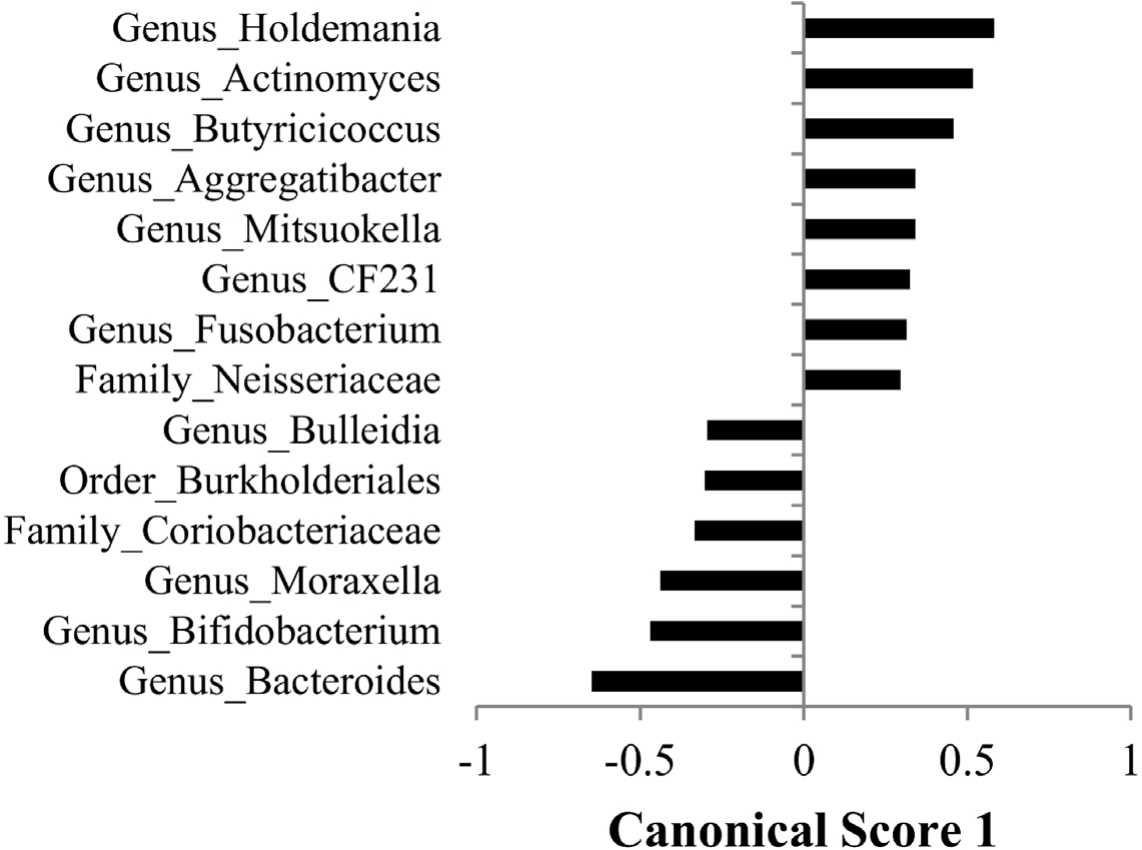
Canonical score 1 for taxa that were found to be significant for the discriminant analysis displayed in Fig 4.

**Fig 6:**
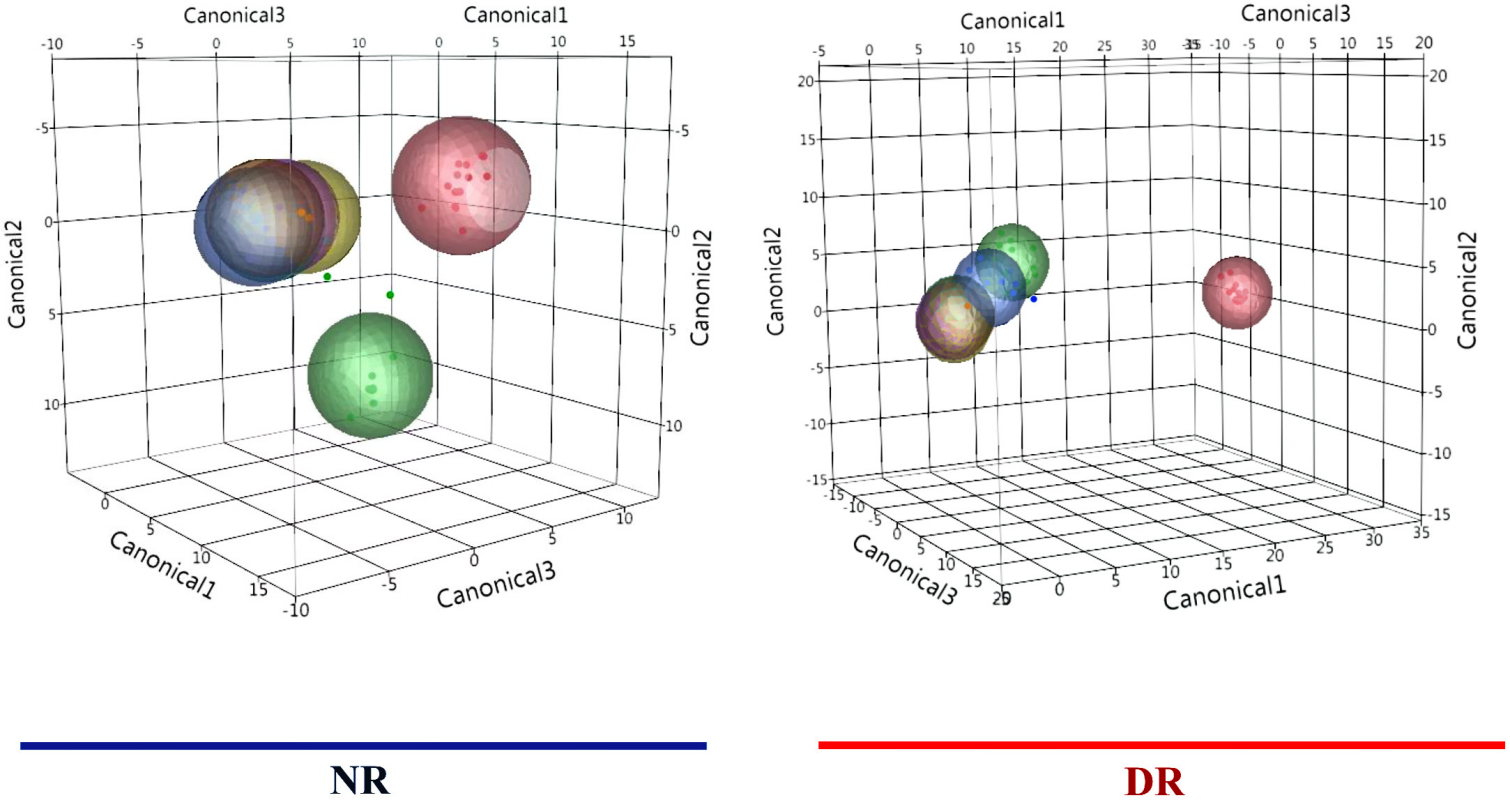
Discriminant analyses of fecal sample microbiome for each treatment group by week. Differences in the fecal microbial profiles for each sampling week (birth = red dots, week one = green dots, week two = blue dots, week three = orange dots, week four = light green dots, week five = purple dots, week six = yellow dots) are illustrated by Canonicals 1, 2 and 3. An ellipse indicates the 95% confidence region to contain the true mean of the group, and a plus symbol indicates the center (centroid) of each group.

**Fig 7:**
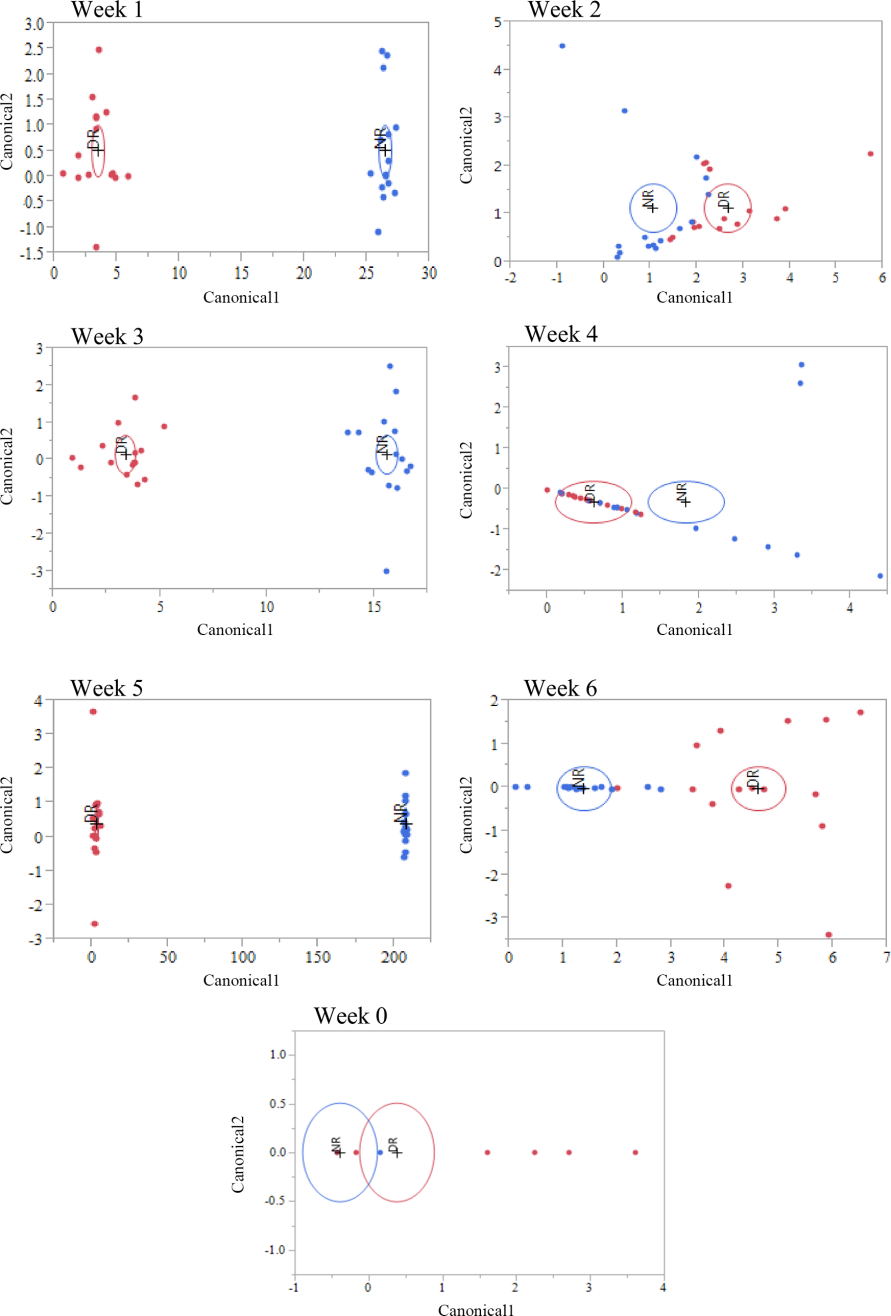
Discriminant analysis for the differences in fecal microbiomes for samples collected from week 1 to 6 by treatment group. Bacteria genus relative abundance was used as covariates and treatment group as the categorical variable (NR = red dots, DR = blue dots). An ellipse indicates the 95% confidence region to contain the true mean of the group, and a plus symbol indicates the center (centroid) of each group.

**Fig 8:**
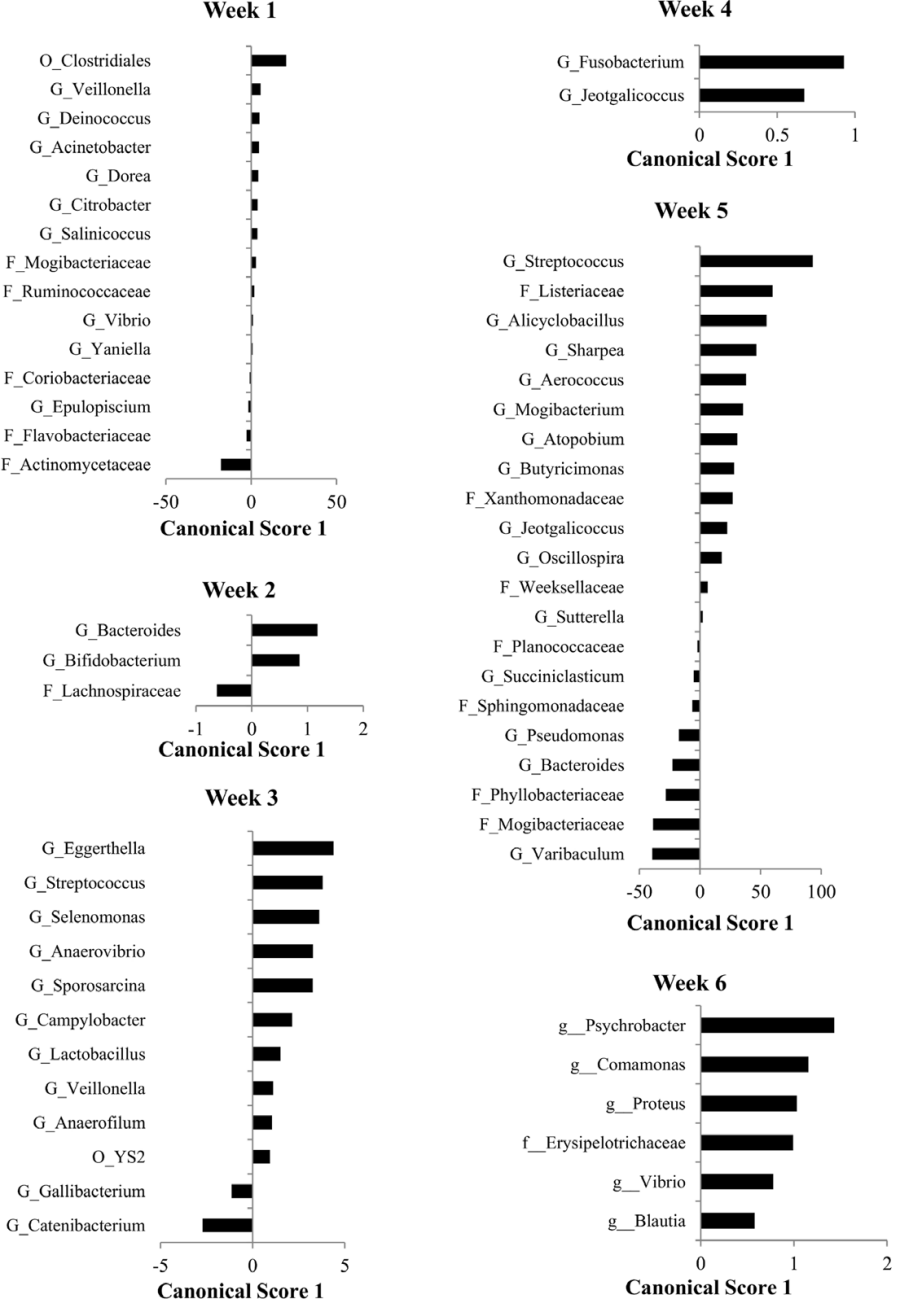
Canonical scores for taxa that were found to be significant for the discriminant analysis displayed in Fig 7.

A discriminant analysis between treatment groups for all sampling weeks combined can be observed in Fig 4, and the Canonical 1 score (difference between DR and NR) can be seen in Fig 5. A discriminant analysis for the microbiota over time in weeks for each treatment group is shown in Fig 6. A discriminant analysis by treatment group for each week is depicted in Fig 7, and the Canonical 1 scores (difference between DR and NR) are displayed on Fig 8.

### Richness, diversity and similarity indexes

Chao 1 richness index was not significantly different between treatment groups. When analyzed within each treatment group, richness index for samples from ND calves varied significantly for weeks 0 (birth), 1 and 4 (S2 Fig). Shannon diversity index was not significantly different between treatment groups for any of the weekly samples.

## Discussion

Our study focused on evaluating the effect of the ingestion of milk containing drugs residues on the fecal microbiota of preweaned dairy calves. The four drugs added to the milk of DR calves were present at the tolerance and safe levels limit for these drugs in raw milk, as established by the Federal Department of Agriculture (FDA). Tolerance levels for drugs are established by the FDA and are deemed as the concentration below which a substance, if present in a food, will have no harmful effects on the human consumers of the food product. From the 210 fecal samples sequenced in our study, discriminant analysis showed that, except for pre-treatment samples (collected at birth before any treatment was administered), calves fed milk with drug residues and calves fed milk without drug residues easily discriminated based on their microbial profile at the genus level for every week following treatment (Fig 7 and Fig 8). This finding showed that consuming drug residues in the milk affected the composition of the microbial population in the feces. However, analysis comparing the abundance of individual taxa between NR and DR showed significant differences only for few genera, with no difference observed at the phylum, class, order and family levels. These results suggest that although drug residues can result in clear discriminant gut microbial communities, they do not result in disruption of taxonomic levels above the genus. This finding suggests that the selective impacts of drug residues present in the milk may exert effects on the competition between microbes predominantly at the strain level, influencing the final balance between these microbial populations.

Discriminant analyses conducted within each treatment group for weekly samples showed that between birth and week 2, calves fed milk without drug residues had a more differentiated microbial profile when compared to calves fed milk containing drug residues (Fig 6). This indicates that drug residues can exert a selection pressure that impacts immature microbiomes that have none or very low resistance to colonization by foreign microbes, what resulted in an abrupt transition to a microbial profile that is most commonly found in older preweaned calves. Evidence for this can be observed by a significantly higher richness index at week 1 when compared to week 0 (birth) for calves in the NR group (**S2 Fig**). For calves in DR group, richness index did not vary significantly between week 0 and week 1, or for any other weekly sampled.

A main disturbance to the microbiota observed after therapeutic exposure to antimicrobials is substantial growth inhibition or death of microbes susceptible to the drug. One study conducted with human patients evaluated the effect of treatment with fluoroquinolones and β-lactams on the fecal microbiota[38]. In that study they collected samples prior to and one week after treatment with these drugs, and observed that fluoroquinolones and β-lactams significantly decreased microbial diversity by 25% and reduced the core phylogenetic microbiota from 29 to 12 taxa. Another study that examined the distal gut microbiota of three individuals over a 10 month span after treatment with ciprofloxacin (a fluoquinolone drug) observed that, although the composition of the gut microbiota stabilized by the end of the experiment, it was altered from its initial state, having an incomplete recovery to the initial state[39]. In our study, exposure of the microbiota to very low antimicrobial drug concentrations did not seem to have a major effect on richness and diversity of the microbiota, with no significant differences observed between the microbiota of samples collected weekly from NR and DR calves (Fig 3). After exposure of a microbe to drug concentrations above the minimum inhibitory concentration (MIC), such as treatment with therapeutic doses of antimicrobial drugs, microbial populations susceptible to the drug will usually be quickly and easily outcompeted by the resistant microbes, even if they carry resistance genes that have a high fitness cost. The fitness cost of a resistance gene is typically measured by comparing the growth rate of isogenic microbes that differ only by the presence of a resistance gene, under culture conditions that do not contain the antimicrobial drug to which the gene confers resistance. Because of the fitness cost frequently associated with resistance to antimicrobial drugs used in medicine, after drug use is stopped and the selective advantage for the resistant microbes is removed, susceptible strains typically will out-compete resistant strains for most microbes. This process is called the classical window for selection of resistance.

Antimicrobial drugs fed in the milk of dairy calves were present a concentration many times below the MIC of important enteric bacteria. As an example, the concentration of tetracycline, ceftiofur, and ampicillin respectively in the milk of DR calves was 53, 80, and 3200 times below the MIC breakpoint for resistant *E*. *coli* [40]. Although the disruption of the microbiota was not as severe as observed with exposure to drugs at or above the MIC, feeding drug residues in the milk clearly discriminated microbial populations between samples obtained from each treatment group (Fig 8). Contrary to the classical selective window discussed above, when microbes are exposed to sub-MICs of drugs the fitness cost of resistance genes is important not only after the exposure to drugs is stopped, but also during the time of exposure. This is due to the fact that sub-MICs of antimicrobials will not completely cripple or kill susceptible bacteria, but instead will only add a burden to their growth, resulting in a small advantage for microbes that carry resistance determinants that do not have a high fitness cost. This sub-MIC selection window is commonly called the minimum selective concentration (MSC), and is defined as the lowest concentration of an antimicrobial drug that still selects for a given resistance determinant [41]. In other words, MSC is the lowest concentration of a drug where carrying a resistance determinant can provide a competitive advantage for a resistant microbe when compared to a susceptible isogenic counterpart. Because of the critical importance of fitness cost under sub-MICs of antimicrobials, the presence of a resistance determinant will not always necessarily result in a lower MSC for the resistant bacteria. For example, in a study by Gullberg et al. (2011), antimicrobial sensitivity tests were conducted for ciprofloxacin using a pair of isogenic *E*. *coli* with either the mutation *gyA*(D87N) or *gyA*(S83L) [42]. Although both these mutations select for resistance to ciprofloxacin, the MSC for *E*. *coli* with *gyA*(D87N) and *gyA*(S83L) was respectively 0.0025 ug/ml and 0.00001 ug/ml. The explanation for this difference is that the fitness cost for the mutation *gyA*(D87N) is 3%, while for *gyA*(S83L) it is 0.2%. Compared to the classical window for selection of resistance, the sub-MIC selection window has a much higher competitive environment, which results in different consequences from the exposure of microbes to these conditions.

A major difference in the sub-MIC selective window is the potential for the selection of a sub-population of strains carrying resistance with low fitness cost that could contribute to their persistence after exposure to an antimicrobial drug ceases. Although the 16S rRNA sequencing used in this study is an effective tool to achieve a glimpse into the phylogenetic diversity of uncultured organisms, its lowest limit of detection is the genus. Moreover, these limitations must be recognized because the effect of sub-MICs of drugs on the microbiota may be occurring at lower taxa than genus (e.g. species and strain). Evidence for the hypothesis that sub-MICs of drugs affect taxa lower than genus can be found in a recent study conducted by our research group, where *Escherichia coli* was isolated from the same fecal samples used in this study, and treatment of calves with drug residues resulted in a significantly higher proportion of multidrug resistant *E*. *coli* when compared to calves being fed milk without addition of drug residues [9].

Abundance of individual genus for all samples collected after calves received the first milk with drug residues (excluding sample collected at birth) revealed that calves receiving drug residues in the milk had significantly lower abundance of Gram-positive bacteria belonging to the *Clostridium* and *Streptococcus* genera when compared to calves receiving milk without drug residues. The antibacterial activity of the cocktail of antimicrobial drugs added to the milk of calves included 1 drug with a narrower activity spectrum covering mostly Gram-positive bacteria (penicillin) and three drugs having a wide range of activity (both Gram-positive and Gram-negative bacteria). Of the drugs added to the milk, tetracycline and penicillin are used at sub-MIC levels as a growth promoting antimicrobial (GPA) in the feed of cattle at concentrations respectively of 44 μg/ml and 0.5 μg/ml [43,44]. Moreover, the GPA concentrations of tetracycline and penicillin are respectively 146 and 100 times above the concentration that these drugs were added to the milk of calves in the DR group. A study in swine evaluating the effect of feeding GPA showed that when pigs were fed GPA with chlortetracycline, sulfamethazine, and penicillin there was a significant reduction in the abundance of *Streptococcus spp*. [45]. A study in broilers feeding GPA containing flavophospholipol (active primarily against Gram-positive bacteria) showed a significant reduction in the number of *Clostridium perfringens* excreted in the feces [46]. Furthermore, the lower abundance of *Streptococcus spp*. and *Clostridium spp*. when exposed to GPA drugs and residual concentration of drugs, as used in our study, suggest that these two bacteria are highly sensitive to the stress of exposure to sub-MICs and/or to environmental changes caused by sub-MICs. Further studies focusing on these specific bacteria would be needed to fully identify what factors are playing a role in the growth rate and death of these bacteria.

Gram-negative *Veillonella* was the only genus that significantly differed in abundance between treatment groups for individual weeks, with a lower prevalence in calves fed drug residues in the milk at week 1 (S1 Fig). *Veillonella spp*. use lactic acid produced by other carbohydrate fermenting bacteria, such as Streptococcus and Lactobacillus, which mean abundance at week 1 was higher in ND samples than in DR samples (although not significantly different between treatment groups). Lack of nutrients for the growth of *Veillonella* bacteria is a potential cause for the lower abundance in DR calves. Another hypothesis is related to the findings of a study that profiled the gut microbiota of children with irritable bowel syndrome (IBS), a functional bowel disorder associated with low-grade inflammation in the mucosal compartment of the gut [47,48]. A notable observations in their study was a significantly lower abundance of *Veillonella spp*. in the gut of IBS children when compared to healthy children. Although GPA has been used for many years in food animals, the exact mechanism for growth enhancement is not clearly understood. Among current hypotheses is that the beneficial effects of GPA in food animals result from inhibition of the production and excretion of catabolic mediators by intestinal inflammatory cells, reducing the expenditure of energy on inflammation [49]. Oxytetracycline, one of the drugs added to DR milk, is known to exhibit multiple significant anti-inflammatory actions, such as inhibition of chemotaxis, granuloma formation, and protease [50]. This anti-inflammatory property would be specifically relevant for calves 1 to 2 weeks of age, when calves are known to be more vulnerable to gastro-intestinal pathogens that could induce inflammation in the gut [51]. Based on these assumptions, feeding milk containing drug residues could potentially have similar anti-inflammatory outcomes as hypothesized for GPA, which could affect the composition of the microbiota and explain the higher abundance of *Veillonella* in calves with higher occurrence of inflammation, as observed in children with IBS.

## Conclusion

The current study used a controlled feeding trial to evaluate the effect of ingesting milk containing drug residues on the fecal microbiota. The microbiota of preweaned calves fed milk with drug residues easily discriminated at the genus level in their weekly microbial profile. Nevertheless, significant relative abundance differences between treatment groups were only observed at the genus level, but not at the phylum, class, order or family level. These results suggest that although drug residues can result in clear discriminant gut microbial communities, it does not result in disruption of taxonomic levels above the genus. Furthermore, future studies on the effect of drug residues on the microbiota should focus on evaluating the composition and function of the microbes and microbiota using methods that allow a more complete evaluation at and below the genus level.

## Supporting Information

S1 Fig Mean relative abundance of the genus *Veillonella spp* for each treatment group by week. **Error bars correspond to a 95% confidence interval.** * Weeks where mean relative abundance was significantly different between treatment groups. Week 0 is the sample collected from calves at birth, prior to receiving any treatment.
(PDF)

S2 Fig Bar graph illustrating the mean Chao richness index and Shannon diversity index for each treatment group by week. Error bars correspond to a 95% confidence interval. Different letters between sampling weeks indicate time points within each treatment group when means were statistically different. Week 0 is the sample collected from calves at birth, prior to receiving any treatment.
(PDF)

S1 Table Mean relative abundance for the 5 most common phyla for each sampling week by treatment group. Week 0 is the sample collected from calves at birth, prior to receiving any treatment.
(PDF)
