# Supplementary material for: Ingestion of Milk Containing Very Low Concentration of Antimicrobials: Longitudinal Effect on Fecal Microbiota Composition in Preweaned Calves
Source: PLoS One. 2016 Jan 25;11(1):e0147525. doi: 10.1371/journal.pone.0147525 (PMC4726667; doi:10.1371/journal.pone.0147525)

S1 Figure. **Mean relative abundance of the genus *Veillonella spp* for each treatment group by week. Error bars correspond to a 95% confidence interval.** * Weeks where mean relative abundance was significantly different between treatment groups. Week 0 is the sample collected from calves at birth, prior to receiving any treatment.

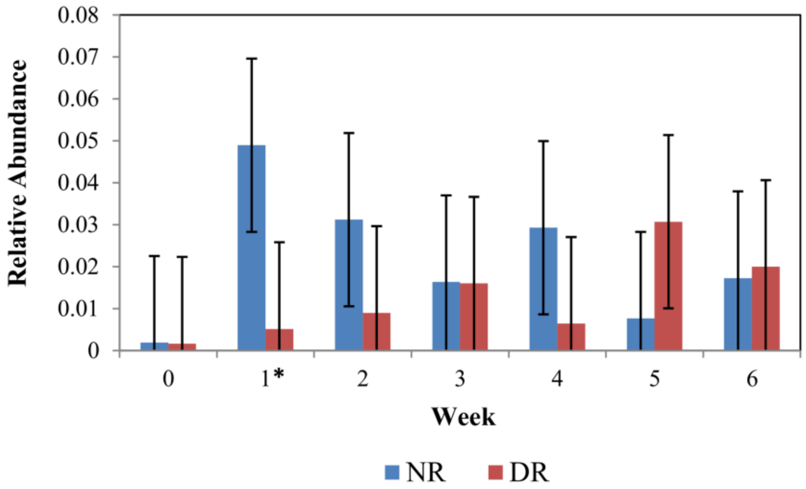

Supplement: S1 Fig — Error bars correspond to a 95% confidence interval. * Weeks where mean relative abundance was significantly different between treatment groups. Week 0 is the sample collected from calves at birth, prior to receiving any treatment. (PDF) [file pone.0147525.s001.pdf]
