# Supplementary material for: Ingestion of Milk Containing Very Low Concentration of Antimicrobials: Longitudinal Effect on Fecal Microbiota Composition in Preweaned Calves
Source: PLoS One. 2016 Jan 25;11(1):e0147525. doi: 10.1371/journal.pone.0147525 (PMC4726667; doi:10.1371/journal.pone.0147525)
Supplement: S1 Table — Week 0 is the sample collected from calves at birth, prior to receiving any treatment. (PDF) [file pone.0147525.s003.pdf]

S3 Table. Mean relative abundance for the 5 most common phyla for each sampling week by treatment group. Week 0 is the sample collected from calves at birth, prior to receiving any treatment.

Time in weeks	Firmicutis			Actinobacteria			Bacteroidetes			Proteobacteria			Verrucomicrobia		
	Mean*		*P*-value**	Mean*		*P*-value**	Mean*		*P*-value**	Mean*		*P*-value**	Mean*		*P*-value**
	DR	NR		DR	NR		DR	NR		DR	NR		DR	NR	
**0**	0.25	0.21		0.02	0.03		0.02	0.04		0.54	0.51		0.002	0.02	
**1**	0.33	0.35		0.32	0.28		0.14	0.07		0.17	0.23		0.0002	0.01	
**2**	0.35	0.43		0.31	0.25		0.19	0.06		0.07	0.14		0.03	0.08	
**3**	0.46	0.51	0.8	0.21	0.2	0.9	0.19	0.09	0.4	0.02	0.06	0.8	0.08	0.1	0.9
**4**	0.45	0.54		0.25	0.18		0.14	0.15		0.01	0.01		0.11	0.08	
**5**	0.52	0.48		0.12	0.12		0.23	0.2		0.01	0.006		0.07	0.11	
**6**	0.43	0.47		0.14	0.1		0.2	0.19		0.01	0.007		0.17	0.18	

*Least Square Mean for the relative abundance for the phylum in question for each week.
** *P*-value testing the hypothesis of a significant difference between treatment groups for the relative abundance for the phylum in question for any of the weeks sampled.
